# Identifying dyspepsia in the Greek population: translation and validation of a questionnaire

**DOI:** 10.1186/1471-2458-6-56

**Published:** 2006-03-04

**Authors:** Foteini Anastasiou, Nikos Antonakis, Georgia Chaireti, Pavlos N Theodorakis, Christos Lionis

**Affiliations:** 1 Clinic of Social and Family Medicine, Department of Social Medicine, Faculty of Medicine, University of Crete, Greece, P.O. Box: 2208, 71003, Heraklion, Crete, Greece; 2 Health Centre of Anogia, 74051 Anogia Crete, Greece; 3 Health Centre of Agia Barbara, 70003, Agia Barbara, Crete, Greece

## Abstract

**Background:**

Studies on clinical issues, including diagnostic strategies, are considered to be the core content of general practice research. The use of standardised instruments is regarded as an important component for the development of Primary Health Care research capacity. Demand for epidemiological cross-cultural comparisons in the international setting and the use of common instruments and definitions valid to each culture is bigger than ever. Dyspepsia is a common complaint in primary practice but little is known with respect to its incidence in Greece. There are some references about the Helicobacter Pylori infection in patients with functional dyspepsia or gastric ulcer in Greece but there is no specific instrument for the identification of dyspepsia. This paper reports on the validation and translation into Greek, of an English questionnaire for the identification of dyspepsia in the general population and discusses several possibilities of its use in the Greek primary care.

**Methods:**

The selected English postal questionnaire for the identification of people with dyspepsia in the general population consists of 30 items and was developed in 1995. The translation and cultural adaptation of the questionnaire has been performed according to international standards. For the validation of the instrument the internal consistency of the items was established using the alpha coefficient of Chronbach, the reproducibility (test – retest reliability) was measured by kappa correlation coefficient and the criterion validity was calculated against the diagnosis of the patients' records using also kappa correlation coefficient.

**Results:**

The final Greek version of the postal questionnaire for the identification of dyspepsia in the general population was reliably translated. The internal consistency of the questionnaire was good, Chronbach's alpha was found to be 0.88 (95% CI: 0.81–0.93), suggesting that all items were appropriate to measure. Kappa coefficient for reproducibility (test – retest reliability) was found 0.66 (95% CI: 0.62–0.71), whereas the kappa analysis for criterion validity was 0.63 (95% CI: 0.36–0.89).

**Conclusion:**

This study indicates that the Greek translation is comparable with the English-language version in terms of validity and reliability, and is suitable for epidemiological research within the Greek primary health care setting.

## Background

Dyspepsia is a common complaint in primary health care (PHC) in most western countries, accounting for 5% of all consultations in general practice [1]. Studies in Europe have reported incidence rates for functional dyspepsia between 8 per 1000 person-years [2] to 13 per 1000 person-years [3]. In Greece there are some hospital-based data on the prevalence of Helicobacter Pylori infection [4,5] but primary care data are lacking. A project on measuring the frequencies of functional gastrointestinal disorders was established on Crete in 2001 and the need of an instrument practical for researchers and PHC physicians for the identification of dyspepsia in Greece was considered a priority. A thorough literature search did not reveal any specific instrument in the Greek language, with the exception of one that refers predominantly to the identification of functional bowel disease [6].

Several instruments have been developed for the identification of dyspepsia [7-10] and its impact on quality of life [11,12]. The English postal questionnaire for the Identification of Dyspepsia in the General Population (IDGP), which was developed and standardised in 1995 by T. Kennedy and R. Jones [10] was considered as appropriate for our purpose for certain reasons; it was developed for the general population; it was short in length and easy to answer (Yes/ No); that meant practical for use in everyday practice. According to the developers it was proved to be accurate and reliable in identifying people with dyspeptic symptoms. The questionnaire had been successfully used in a UK population study for the prevalence of gastro-esophageal reflux disease (GERD) symptoms [13].

This paper reports on the translation and validation of the IDGP and discusses several possibilities of its use in the Greek primary care.

## Methods

### Questionnaire

The original questionnaire consists of 8 short questions on demographics and a core part of 30 items, 29 of which are answered by Yes or No. An open question at the end of the questionnaire gives an opportunity for the patient to refer to what ever seems important for the matter and was not asked (Additional file 1). The IDGP classifies the symptoms into clinical subgroups namely dyspepsia, GERD like symptoms, past diagnosis of peptic ulcer. According to the questionnaire dyspepsia is diagnosed by the presence of "any of the symptoms of dyspepsia in the last year" [10]. GERD is likely when either heartburn or acid regurgitation is present also in the last year. Furthermore, the IDGP seeks the frequency of the dyspeptic and GERD like symptoms along with patients' consultation behaviour. The questionnaire proved to have a good internal consistency (an overall kappa coefficient 0.92) [10].

### Translation

The translation and cultural adaptation of IDGP were performed according to "The Minimal Translation Criteria" [14]. Two independent bilingual physicians forward translated the questionnaire; two other physicians, native English speakers, then back translated the agreed Greek version. The agreed back translation was sent to the authors of the original questionnaire for comparison and their suggestions were incorporated into the final Greek version.

A cognitive debriefing process was then used for the cultural adaptation of the questionnaire [14]. This process was carried out in order to identify any areas presenting problematic language, and to assess the patient's level of understanding.

The questionnaire was administered to five attendants of a PHC centre, and comments made by them were discussed and included to the final Greek version.

### Validation

Reliability was assessed by measuring internal consistency and reproducibility (test- retest reliability) [15,16]. Internal consistency was determined by checking the components of a questionnaire against each other, using Chronbach's alpha [17-19].

A minimum value of 0.70 for group and 0.90 for individual comparisons is generally desirable [19,20].

Reproducibility (test- retest reliability) is a measure of strength of association for determining stability of the questionnaire's results over time because it corrects for lack of independence between measurement intervals [15]. Forty consecutive PHC attendants visiting one rural PHC unit in Crete over a period of two months were recruited and asked to complete the questionnaire twice with an interval of 3 weeks. All participants had a record of upper abdominal symptoms during the past year; no one refused to complete the questionnaire. The overall Cohen's kappa coefficient was estimated [16].

Criterion validity refers to the extent to which the instrument correlates with a gold standard [21]. To define the criterion validity of the questionnaire, the diagnoses available in medical records of a fully qualified General Practitioner (GP) of the rural PHC unit were used as a gold standard to which we compared the outcome of the questionnaire given on the first visit. Kappa analysis was used in order to assess agreement between the diagnoses (dyspepsia / GERD or ulcer) as they were confirmed by the questionnaires and the GP. The diagnose of dyspepsia in our validation process was established according to the Rome II definition [22] by the positive answer to one or more of items 1, 4 or 18, (pain or discomfort, feeling of excess wind or fullness, nausea) combined with negative response on the items referring to GERD like symptoms. The diagnosis of GERD was made by the positive response to one of the items 7, 10, 13 and 15 (heartburn, heart burn when lying in bed, heartburn only when lying in bed, acid tasting fluid at the back of the throat). Ulcer was diagnosed when there was a positive answer to item 27 (past diagnosis of stomach or duodenal ulcer).

A factor analysis was performed in order to identify the separate factors, which make-up this questionnaire and highlight how the items group together [23]. Factor structure was studied by Principal Component Analysis using Varimax with Keiser Normalization as Rotation Method. Both Kaiser criteria for applicability were fulfilled [24]. An analysis on the patients' symptoms (items 1, 4, 7, 10, 13, 14, 15, 18, 21, 24) was performed and a factor was considered as important if its eigenvalue value exceeded 1.0 [23].

### Ethics

The scientific committee of the University Hospital of Heraklion, Crete has approved this study (number of protocol: 7173/ 12/7/2000). All participants in the cultural adaptation and reproducibility (test- retest reliability) procedure were informed about the scope and the purpose of the study and provided their oral consent.

## Results

### Translation

The authors suggested changes to the demographic data section of the questionnaire and added questions regarding employment. They further suggested making all items referring to the duration of the symptom(s) more specific by replacing the phrase "the past year" with the phrase "the last 12 months" in accordance with the latest definitions of Rome II [22]. The concept of discomfort was also taken into account, and the word "discomfort" was added also to the second question according to the same criteria.

During the process of cultural adaptation only one of the five patients reported problems in comprehension of the questionnaire in the total. Problems were focused mostly in expressions used and less in the understanding of the actual questions.

The two older and less educated participants reported some problems but any misunderstanding was solved after they read again the troubling question. No external help was given to the participants regarding the meaning of any of the questions. The suggestion of a bigger picture was accepted as well as the suggestion to explain in parenthesis the areas shown in the picture (Additional file 2).

### Validation

The IDGP questionnaire showed a high overall internal consistency (alpha value: 0.88, 95% CI: 0.81–0.93) for individual comparison. Each diagnostic group also showed acceptable alpha values: 0.81 for dyspepsia; 0.76 for frequent dyspepsia; 0.82 for GERD like symptoms; 0.75 for frequent GERD like symptoms; 0.89 for investigation for organic gastric disease; 0.82 for past diagnosis of stomach or duodenal ulcer, while internal consistency was relatively low for consultation behaviour: 0.66 and for the open question: 0.72.

The overall Cohen's kappa coefficient for the reproducibility (test – retest reliability) of the questionnaire was found "substantial" (0.66, 95% CI: 0.62–0.71) [16]. Twenty-five of the 30 items had good reproducibility (Cohen's kappa coefficient>0.40), while the remaining five items had a fair reproducibility (Cohen's kappa coefficient<0.40). These results are illustrated in Table 1.

The kappa coefficient for criterion validity was also "substantial" (0.63, 95% CI: 0.36–0.89) and the overall agreement between the results of the questionnaire and the doctor's diagnose was 85%.

The performed factor analysis indicated three factors with eigenvalue over 1.0. Those factors were responsible for 61,34 % of variance and rotation converged in 4 iterations (Table 2).

## Discussion

The development of academic general practice within the Mediterranean setting does not only need support and funds but also research capacity [25]. Studies on "clinical issues", including diagnostic strategies, are considered to be the core content of general practice research as a recent publication reported [26]. Thus, the use of standardised instruments is considered as an important component for the development of PHC research capability and some questionnaires measuring the frequency of health problems in primary care and the impact of ill conditions in quality of life of Greek patients have been already published [27,28]. Moreover, the increasing demand for epidemiological cross-cultural comparisons in the international setting and the use of common instruments and definitions valid to each culture is stronger than ever [21].

We focused on dyspepsia because it is a symptom with which patients frequently present to PHC services worldwide. In addition, no data regarding the prevalence of dyspepsia in primary care population in Greece have been reported. We followed international criteria for the translation, and the Greek version was well perceived by the participants in the pilot study. The validation process revealed a "substantial" Cohen's kappa coefficient for the questionnaire and the satisfactory Chronbach's alpha suggests that the instrument is reliable for the Greek setting. The criterion validity was also good supporting that our instrument was valid when we judged it with the diagnosis of the GP as a gold standard. The factor analysis of the symptoms revealed the shared variance of 3 separate factors.

However, there are some concerns in terms of its validation into Greek language and particularly: (a) in some questions reproducibility (test – retest reliability) was found to be fair to moderate. Those questions referred mostly to consultation behaviour and did not change the outcome of the questionnaire, thus they were not considered as a strong limitation for the use of the instrument.

(b) during the reproducibility (test – retest reliability) process patients were informed that they would be invited sometime in the future to answer the questionnaire for a second time. It was unavoidable for us to not disclosure this issue when we were seeking for permission and making aware the respondent about the scope of the study. However patients did not know when they would be asked again.

(c) the original questionnaire was developed prior to the Rome II consensus. Nevertheless it is approaching the Rome II definition of dyspepsia and the modified Greek version is much more closer to Rome II consensus.

(d) overlap with IBS is potential since there is no question referring to the bowel habits. The simultaneous use with an IBS specific instrument or a combined questionnaire for both diseases [29] is recommended.

(e) item 4 that refers to the "feeling of excess wind or fullness" is generally accepted as a symptom which is included in the dyspepsia definition, however in the factor analysis a potential overlap with the GERD like symptoms is indicated.

The translated and validated questionnaire is anticipated to be a practical instrument for primary care physicians in Greece; it can be applied in daily practice for identifying patients with dyspepsia. Greek speaking doctors who are practicing in Cyprus and other countries may find it helpful and the questionnaire could be used in epidemiological studies highlighting some of the missing information from Greece.

## Conclusion

In conclusion, the Greek translated questionnaire appears to be a reliable and valid tool for the identification of dyspepsia in clinical practice. Due to its short length and consumption of time it seems to be a practical instrument in the Greek primary care.

## List of Abbreviations

PHC: Primary Health Care.

IDGP: Identification of Dyspepsia in the General Population questionnaire.

GERD: Gastro-esophageal reflux disease.

GP: General Practitioner.

## Competing interests

The author(s) declare that they have no competing interests.

## Authors' contributions

CL conceived the study design, participated in the translation of the questionnaire, formed the layout of the manuscript and wrote the final draft of the manuscript.

FA participated in the translation of the questionnaire, contributed in the data collection, carried out the analysis and co- wrote the final manuscript.

NA carried out the statistical analysis and co- wrote the final manuscript.

GH participated in the data collection and interpretation.

PNT contributed in the data interpretation and the final manuscript.

All authors approved the final manuscript.

## Pre-publication history

The pre-publication history for this paper can be accessed here:



## Supplementary Material

Additional File 1 **The original English questionnaire**. The original English questionnaire. Click here for file

Additional File 2 **The Greek version of the questionnaire**. The final Greek version of the questionnaire. Click here for file

## Figures and Tables

**Table 1:** IDGP: Reproducibility (test- retest reliability).

DIAGNOSTIC CATEGORIES	K^+^	ITEM	K^+^
Dyspepsia	0.67	1	0.724
		4	0.609
		18	0.603
Frequent dyspepsia	0.61	2	0.358
		5	0.694
		19	0.691
GERD like symptoms	0.69	7	0.746
		10	0.694
		13	0.314
		14	0.730
		15	0.652
		21	0.658
		24	0.749
Frequent GERD like symptoms	0.71	8	0.700
		11	0.742
		16	0.698
		22	0.444
Consultation behaviour	0.49	3	0.413
		6	0.405
		9	0.336
		12	0.481
		17	0.688
		20	0.306
		23	0.278
		26	0.722
Investigation for organic gastric disease	0.80	28	0.653
		29	0.950
Past diagnosis of stomach or duodenal ulcer		27	0.688
Open question		30	0.615

^+^: Kappa coefficient

**Table 2:** Factor analysis for the symptoms: Rotated Component Matrix for 3 factors.

	Component		
SYMPTOMS	1	2	3

(Item 1) Pain or discomfort			0.870
(Item 4) Feeling of excess wind or fullness in the upper abdomen	0.566		
(Item 7) Heartburn	0.777		
(Item 10) Heartburn when lying in bed	0.882		
(Item 13) Heartburn only when lying in bed	0.483		
(Item 14) Awakened by the heartburn	0.861		
(Item 15) Acid taste at the back of the throat	0.555		
(Item 18) Nausea		0.816	
(Item 21) Vomiting		0.876	
(Item 24) Difficulty in swallowing	0.651		
			
Eigenvalues	3.60	1.40	1.13
Degree of explanation (%)	36.00	14.03	11.32
