# Supplementary material for: Identifying dyspepsia in the Greek population: translation and validation of a questionnaire
Source: BMC Public Health. 2006 Mar 4;6:56. doi: 10.1186/1471-2458-6-56 (PMC1420284; doi:10.1186/1471-2458-6-56)
Supplement: Additional File 1 — The original English questionnaire. The original English questionnaire. [file 1471-2458-6-56-S1.doc]

THANK YOU FOR YOUR WILLINGNESS TO HELP

TO ANSWER THE QUESTIONS PLACE A TICK IN THE APPROPRIATE BOX

IF YOU ARE UNSURE OF THE ANSWERS TICK “NO”.

Kindly return the questionnaire in the FREEPOST envelope. No stamp is required.

What is your…?

Name: ___________________________________________ Age: _____ Sex: Male Female

Address: ________________________________________________________________

________________________________________________________________

Post code: Telephone number: _____________________

What is/was your occupation: _______________________________________________

What is your education:

What is/was your partner’s occupation: ________________________________________

Are you:

Employed full time? Housewife?

Part time? Retired?

Unemployed? Student?

REMEMBER, IF YOU UNSURE OF THE ANSWER TO ANY OF THE QUESTION BELOW, THEN TICK “NO”

Have you had pain or discomfort in the place shown

in the picture in the last year? Yes No

If Yes to the last question then:

Have you had this pain or discomfort?

On more than six occasions in the last year? Yes No

Did you see a doctor about it? Yes No

Have you had a feeling of excess wind or fullness in the place shown

in the picture after eating or drinking in the last year? Yes No

If Yes to the last question then:

Have you had this feeling on more than six occasions in the last year? Yes No

Did you see a doctor about it? Yes No

Heartburn is a burning or ache behind the breast bone in the

chest, that is not due to angina or heart trouble.

Have you had heartburn in the last year? Yes No

If Yes to the last question then:

Have you had this feeling on more than six occasions in the last year? Yes No

Did you see a doctor about it? Yes No

When lying in bed have you had heartburn during the last year? Yes No

If Yes then:

Has this happened on more than six occasions in the last year? Yes No

Did you see a doctor about it? Yes No

Do you get heartburn **only** when lying in bed? Yes No

Does this heartburn waken you from your sleep? Yes No

Have you had a very sour or acid tasting fluid at the back of your

throat in the last year? Yes No

If Yes:

Has this happened on more than six occasions in the last year? Yes No

Did you see a doctor about it? Yes No

Have you had a feeling of wanting to throw out (nausea),

in the last year? Yes No

If Yes:

Has this happened on more than six occasions in the last year? Yes No

Did you see a doctor about it? Yes No

Have you actually thrown up (vomited) in the last year? Yes No

If Yes:

Has this happened on more than six occasions in the last year? Yes No

Did you see a doctor about it? Yes No

Have you had difficulty swallowing (food sticking in you throat)

in the last year? Yes No

If Yes:

Has this happened on more than six occasions in the last year? Yes No

Did you see a doctor about it? Yes No

Have you ever been diagnosed as having a gastric (stomach)

or duodenal ulcer? Yes No

Have you ever had a barium meal examination? Yes No

(You have to drink a white liquid before the X-rays are taken)

Have you ever had an endoscopy or gastroscopy? Yes No

(A tube with a light source is swallowed to look

inside the stomach)

**Is there anything, which we have not asked about, and which you think would be important for us to know?**

THANK YOU ONCE AGAIN FOR YOUR GENEROUS ASSISTANCE.

KINDLY CHECK THAT ALL QUESTIONS ARE ANSWERED, EVEN THE “NO” ONES, THEN PLEASE RETURN THE QUESTIONNAIRE IN THE FREEPOST ENVELOPE.
