# Supplementary material for: Identifying dyspepsia in the Greek population: translation and validation of a questionnaire
Source: BMC Public Health. 2006 Mar 4;6:56. doi: 10.1186/1471-2458-6-56 (PMC1420284; doi:10.1186/1471-2458-6-56)
Supplement: Additional File 2 — The Greek version of the questionnaire. The final Greek version of the questionnaire. [file 1471-2458-6-56-S2.doc]

ΕΥΧΑΡΙΣΤΟΥΜΕ ΓΙΑ ΤΗΝ ΣΥΓΚΑΤΑΘΕΣΗ ΣΑΣ ΝΑ ΒΟΗΘΗΣΕΤΕ.

ΓΙΑ ΝΑ ΑΠΑΝΤΗΣΕΤΕ ΤΙΣ ΕΡΩΤΗΣΕΙΣ ΤΟΠΟΘΕΤΗΣΤΕ ΕΝΑ Χ ΣΤΟ ΚΑΤΑΛΛΗΛΟ ΤΕΤΡΑΓΩΝΑΚΙ ΕΑΝ ΔΕΝ ΕΙΣΤΕ ΣΙΓΟΥΡΟΙ ΓΙΑ ΤΙΣ ΑΠΑΝΤΗΣΕΙΣ ΣΗΜΕΙΩΣΤΕ ΤΟ “ΟΧΙ”

Επιστρέψτε το ερωτηματολόγιο με τον φάκελο που εσωκλείεται. Δεν απαιτείται γραμματόσημο.

Παρακαλώ συμπληρώστε τα στοιχεία σας:

Ονοματεπώνυμο : ηλικία: φύλο: Άρρεν

Θήλυ

Διεύθυνση:

Ταχ.Κωδ. Αριθ.Τηλ.

Ποιο είναι/ ήταν το επάγγελμα σας;

Ποια είναι η εκπαίδευση σας;

Ποιο είναι/ ήταν το επάγγελμα του συντρόφου σας;

Είστε εργαζόμενος /η :

Πλήρους απασχόλησης; Νοικοκυρά; Ελεύθερος επαγγελματίας;

Μερικής απασχόλησης; Συνταξιοδοτημένος; Υπάλληλος;

Άνεργος/ η; Μαθητής/ Φοιτητής;

**ΘΥΜΗΘΕΙΤΕ ΟΤΙ ΕΑΝ ΔΕΝ ΕΙΣΤΕ ΣΙΓΟΥΡΟΙ ΓΙΑ ΤΗΝ ΑΠΑΝΤΗΣΗ ΣΕ ΟΠΟΙΑΔΗΠΟΤΕ ΑΠΟ ΤΙΣ ΠΑΡΑΚΑΤΩ ΕΡΩΤΗΣΕΙΣ ΤΟΤΕ ΣΗΜΕΙΩΣΤΕ ΤΟ “ΟΧΙ”**

Είχατε πόνο ή δυσφορία/ ενόχληση στις περιοχές που

υποδεικνύονται στην εικόνα (άνω κοιλία-στομάχι), ΝΑΙ ΟΧΙ

τους τελευταίους 12 μήνες;

Αν Ναι στην τελευταία ερώτηση τότε:

Είχατε αυτόν τον πόνο ή δυσφορία περισσότερο από

έξι φορές τους τελευταίους 12 μήνες; ΝΑΙ ΟΧΙ

Είδατε κάποιο γιατρό για το λόγο αυτό; ΝΑΙ ΟΧΙ

Είχατε ποτέ, ένα αίσθημα φουσκώματος ή πληρότητας

στις περιοχές που υποδεικνύονται στην εικόνα,

μετά τη λήψη τροφής ή ποτού, τους τελευταίους 12 μήνες; ΝΑΙ ΟΧΙ

Αν Ναι στην τελευταία ερώτηση τότε:

Είχατε αυτό το αίσθημα περισσότερο από έξι

φορές τους τελευταίους 12 μήνες; ΝΑΙ ΟΧΙ

Είδατε κάποιο γιατρό για το λόγο αυτό; ΝΑΙ ΟΧΙ

Οπισθοστερνικό καύσος είναι ένα αίσθημα καψίματος ή πόνου πίσω

από το στήθος, που δεν οφείλεται σε στηθάγχη

ή σε καρδιακή διαταραχή.

Είχατε οπισθοστερνικό καύσος τους τελευταίους 12 μήνες; ΝΑΙ ΟΧΙ

Αν Ναι στην τελευταία ερώτηση τότε:

Είχατε αυτό το αίσθημα περισσότερο από έξι

φορές τους τελευταίους 12 μήνες; ΝΑΙ ΟΧΙ

Είδατε κάποιο γιατρό για το λόγο αυτό; ΝΑΙ ΟΧΙ

Όταν ξαπλώνετε στο κρεβάτι είχατε ποτέ οπισθοστερνικό καύσος,

κατά τη διάρκεια των τελευταίων 12 μηνών; ΝΑΙ ΟΧΙ

Αν Ναι τότε:

Συνέβη αυτό περισσότερο από έξι φορές τους τελευταίους 12 μήνες; ΝΑΙ ΟΧΙ

Είδατε κάποιο γιατρό για το λόγο αυτό; ΝΑΙ ΟΧΙ

Έχετε οπισθοστερνικό καύσος **μόνο** όταν ξαπλώνετε στο κρεβάτι; ΝΑΙ ΟΧΙ

Σας ξυπνάει το οπισθοστερνικό καύσος όταν κοιμάστε; ΝΑΙ ΟΧΙ

Είχατε ξινίλα ή γεύση όξινου υγρού στο πίσω τμήμα

του λαιμού σας τους τελευταίους 12 μήνες; ΝΑΙ ΟΧΙ

Αν Ναι: Συνέβη αυτό περισσότερο από έξι φορές

τους τελευταίους 12 μήνες; ΝΑΙ ΟΧΙ

Είδατε κάποιο γιατρό για το λόγο αυτό; ΝΑΙ ΟΧΙ

Είχατε ποτέ κατά τους τελευταίους μήνες το αίσθημα ότι

θέλετε να κάνετε έμετο (ναυτία); ΝΑΙ ΟΧΙ

Αν Ναι: Συνέβη αυτό περισσότερο από έξι φορές

τους τελευταίους 12 μήνες; ΝΑΙ ΟΧΙ

Είδατε κάποιο γιατρό για το λόγο αυτό; ΝΑΙ ΟΧΙ

Έχετε πραγματική εξαγωγή (έμετο) τους τελευταίους 12 μήνες; ΝΑΙ ΟΧΙ

Αν Ναι: Συνέβη αυτό περισσότερο από έξι φορές

τους τελευταίους 12 μήνες; ΝΑΙ ΟΧΙ

Είδατε κάποιο γιατρό για το λόγο αυτό; ΝΑΙ ΟΧΙ

Είχατε δυσκολία να καταπιείτε (τροφή να κολλάει

στο λαιμό σας) τους τελευταίους 12 μήνες; ΝΑΙ ΟΧΙ

Αν Ναι: Συνέβη αυτό περισσότερο από έξι φορές

τους τελευταίους 12 μήνες; ΝΑΙ ΟΧΙ

Είδατε κάποιο γιατρό για το λόγο αυτό; ΝΑΙ ΟΧΙ

Είχατε ποτέ διαγνωσθεί ότι έχετε στομαχικό

ή δωδεκαδακτυλικό έλκος; ΝΑΙ ΟΧΙ

Υποβληθήκατε ποτέ σε εξέταση με βαριούχο γεύμα;

(Πίνετε ένα λευκό υγρό πριν να βγουν οι ακτινογραφίες) ΝΑΙ ΟΧΙ

Υποβληθήκατε ποτέ σε ενδοσκόπηση ή γαστροσκόπηση;

(Ένας σωλήνας με ένα φως καταπίνεται για

να εξετάσει το εσωτερικό του στομάχου) ΝΑΙ ΟΧΙ

**Υπάρχει κάτι για το οποίο δε σας ρωτήσαμε και νομίζετε ότι θα ήταν σημαντικό να μας το πείτε;**

ΕΥΧΑΡΙΣΤΟΥΜΕ ΚΑΙ ΠΑΛΙ ΓΙΑ ΤΗΝ ΠΟΛΥΤΙΜΗ ΒΟΗΘΕΙΑ ΣΑΣ

ΠΑΡΑΚΑΛΟΥΜΕ ΝΑ ΕΛΕΓΞΕΤΕ ΑΛΛΗ ΜΙΑ ΦΟΡΑ ΟΤΙ ΕΧΟΥΝ ΑΠΑΝΤΗΘΕΙ ΟΛΕΣ ΟΙ ΕΡΩΤΗΣΕΙΣ ΑΚΟΜΑ ΚΑΙ ΕΚΕΙΝΕΣ ΜΕ “ΟΧΙ” ΚΑΙ ΜΕΤA ΕΠΙΣΤΡEΨΕΤΕ ΤΟ ΕΡΩΤΗΜΑΤΟΛΟΓΙΟ ΣΤΟ ΦΑΚΕΛΟ ΕΛΕΥΘΕΡΟΥ ΤΑΧΥΔΡΟΜΕΙΟΥ ΠΟΥ ΕΣΩΚΛΕΙΕΤΑΙ

Το ερωτηματολόγιο χρησιμοποιείτε μετά από άδεια των συγγραφέων T. Kennedy και R. Jones.
